# A Synchronization Criterion for Two Hindmarsh-Rose Neurons with Linear and Nonlinear Coupling Functions Based on the Laplace Transform Method

**DOI:** 10.1155/2021/6692132

**Published:** 2021-02-02

**Authors:** Chunlin Su, Bin Zhen, Zigen Song

**Affiliations:** ^1^School of Environment and Architecture, University of Shanghai for Science and Technology, Shanghai 200093, China; ^2^College of Information Technology, Shanghai Ocean University, Shanghai 201306, China

## Abstract

In this paper, an analytical criterion is proposed to investigate the synchronization between two Hindmarsh-Rose neurons with linear and nonlinear coupling functions based on the Laplace transform method. Different from previous works, the synchronization error system is expressed in its integral form, which is more convenient to analyze. The synchronization problem of two HR coupled neurons is ultimately converted into the stability problem of roots to a nonlinear algebraic equation. Then, an analytical criterion for synchronization between the two HR neurons can be given by using the Routh-Hurwitz criterion. Numerical simulations show that the synchronization criterion derived in this paper is valid, regardless of the periodic spikes or burst-spike chaotic behavior of the two HR neurons. Furthermore, the analytical results have almost the same accuracy as the conditional Lyapunov method. In addition, the calculation quantities always are small no matter the linear and nonlinear coupling functions, which show that the approach presented in this paper is easy to be developed to study synchronization between a large number of HR neurons.

## 1. Introduction

The study of neurons plays a very important role in many applications in neural science, brain science, and so on. The famous Hodgkin-Huxley (HH) equation, proposed by Hodgkin and Huxley in 1952, [1, 2] usually was used to construct neural systems or exhibit the neural dynamic behavior. Since then, other neuronal models, such as the FizHugh-Nagumo (FHN) model [3], the Hindmarsh-Rose (HR) model [4], the Chay model [5], and the Morris-Lecar model [6], also have been published. The HR model was constructed from voltage clamp data to provide a simple description of the patterned activity observed in molluscan neurons. Although the HR model is not based on physiology wholly, it can exhibit some features observed in neuronal bursting. The HR model has been investigated from bifurcation analysis to the firing mechanism [7].

Synchronization processes are ubiquitous in nature. Synchronization of coupled networks has been studied with increasing interest over the last few decades due to its numerous potential applications [8–11]. One way to gain a deeper understanding of synchronization in complex networks is to investigate the stability of the completely synchronized state of the population of identical oscillators [12, 13], which has been extensively investigated based on Lyapunov's direct method [14, 15]. One of the most popular and widely used methods to determine stability of the synchronized state is using the Lyapunov exponents as average measurements of expansion or shrinkage of small displacements along synchronized trajectory, which is called the conditional Lyapunov exponent method [16–18].

Pecora and Carroll [19] proposed the master stability function (MSF) method to discuss the local stability of the synchronization manifold. This method provides an objective criterion to characterize the stability of the global synchronized state, independent of particularities of oscillators. Relevant insights about the structure-dynamics relationship have been obtained using this technique. Chen [20] and Li et al. [21] obtained conditions for synchronization using the matrix measure approach. Their criteria do not depend on solutions of the synchronous state and have fewer limitations on network connections. He [22] presented a new approach for chaotic synchronization of HR neural networks with a special nonlinear coupling function. Synchronization can be achieved without the requirement to calculate the maximum Lyapunov exponents when the coupling strengths are taken as reference values. Lu and Chen [23] used a variation method near the projection on the synchronization manifold to analyze synchronization of linearly coupled ordinary differential equations. The uncoupled dynamical behavior at each node is general and can be chaotic or otherwise; the coupling configuration is also general, with the coupling matrix not assumed to be symmetric or irreducible.

The above methods greatly further one's understanding of synchronization in complex networks. However, the results obtained by these methods are limited. Conditions for synchronization obtained by using the Lyapunov function method are often too conservative. It is also difficult to find a proper Lyapunov function. For most cases, calculating the conditional Lyapunov exponents is a cumbersome procedure. It should also be noted that the negativity of the conditional Lyapunov exponents is only a necessary condition for the stability of the synchronized state. Thus, additional conditions should be added to ensure synchronization in a necessary and sufficient way [24]. The MSF method [19] proposed by Pecora and Carroll requires that the Laplacian matrices are diagonal or block diagonal. Nishikawa and Motter [25] developed an extension of the master stability framework to study local synchronization in any symmetric and asymmetric network with any output function. The method has some variations, but generally the stability analysis is embarrassed by the need to calculate the conditional Lyapunov exponents. Using the matrix measure approach requires calculating the Lyapunov exponents of the map to achieve a sufficient condition for synchronization. The new method proposed by He [22] is only valid for a special nonlinear coupling function and coupling strengths. The local stability condition presented in [23] for synchronization is related to the synchronization trajectory. Since the synchronization trajectory is unknown in advance, the criterion is hard to be used in applications. To sum up, in addition to Lyapunov's direct method, nearly all synchronization criteria require the computation of Lyapunov exponents, eigenvalues of the coupling matrix, or the synchronization trajectory, which is inconvenient and ineffective. Therefore, more convenient and effective approaches to determine synchronization need to be developed.

In this paper, we use the Laplace transform to investigate the synchronization conditions for two HR neurons with linear and nonlinear coupling functions. Different from other studies, the synchronization error system derived from the original coupled neural system is converted into sets of Volterra integral equations based on the convolution theorem in the Laplace transform. The synchronization problem of two HR coupled neurons is ultimately converted into the stability problem of roots to a nonlinear algebraic equation. Then, an analytical criterion for synchronization in the two HR neurons can be given by using the Routh-Hurwitz criterion. Numerical simulations show that the synchronization criterion derived in this paper is valid, regardless of the periodic spikes or burst-spike chaotic behavior of the two HR neurons. Additionally, the analytical results have almost the same accuracy as the conditional Lyapunov method. The rest of the paper is organized as follows. In Section 2, the synchronization conditions for two HR neurons with a linear coupling function are discussed by using the Laplace transform. In Section 3, the case of a nonlinear coupling function is investigated. In Section 4, numerical simulations are carried out to verify the effectiveness of the analytical criterion derived in Sections 2 and 3. Conclusions are drawn in Section 5.

## 2. Two HR Neurons with a Linear Coupling Function

A HR neuron is described by the following equation of motion:

(1) $$\begin{array}{c} \dot{x}=y-ax^{3}+bx^{2}-z+I,\\ \dot{y}=c-dx^{2}-y,\\ \dot{z}=rs_{0}\left(x-x_{0}\right)-rz, \end{array}$$

where *x* represents the membrane potential, *y* is a recovery variable associated with fast current, *z* is a slowly changing adaptation current. *a*, *b*, *c*, *d*, *s*_0_, *r*, and *x*_0_ are parameters, and *I* is the external current input. Consider two coupled HR neurons with a linear coupling function:

(2) $$\begin{array}{c} {\dot{x}}_{1}=y_{1}-ax_{1}^{3}+bx_{1}^{2}-z_{1}+I+\beta \left(x_{1}-x_{2}\right),\\ {\dot{y}}_{1}=c-dx_{1}^{2}-y_{1},\\ {\dot{z}}_{1}=rs_{0}\left(x_{1}-x_{0}\right)-rz_{1},\\ {\dot{x}}_{2}=y_{2}-ax_{2}^{3}+bx_{2}^{2}-z_{2}+I+\beta \left(x_{2}-x_{1}\right),\\ {\dot{y}}_{2}=c-dx_{2}^{2}-y_{2},\\ {\dot{z}}_{2}=rs_{0}\left(x_{2}-x_{0}\right)-rz_{2}, \end{array}$$

where *β* is the coupling strength. If ∣*x*_1_ − *x*_2_ | →0, ∣*y*_1_ − *y*_2_ | →0, and ∣*z*_1_ − *z*_2_ | →0 for *t* → ∞, synchronization between two HR neurons is achieved. Assume that (*x*_01_, *y*_01_, *z*_01_) is the equilibrium point of system (1). For simplicity, substituting

(3) $$\begin{array}{c} u_{1}=x_{1}-x_{01},\\ u_{2}=y_{1}-y_{01},\\ u_{3}=z_{1}-z_{01},\\ v_{1}=x_{2}-x_{01},\\ v_{2}=y_{2}-y_{01},\\ v_{3}=z_{2}-z_{01}, \end{array}$$

into equation (2), one has

(4) $$\begin{array}{c} {\dot{u}}_{1}=\phi_{1}u_{1}+\phi_{2}u_{1}^{2}-au_{1}^{3}+u_{2}-u_{3}+\beta \left(u_{1}-v_{1}\right),\\ {\dot{u}}_{2}=\phi_{3}u_{1}-du_{1}^{2}-u_{2},\\ {\dot{u}}_{3}=r\left(s_{0}u_{1}-u_{3}\right),\\ {\dot{v}}_{1}=\phi_{1}v_{1}+\phi_{2}v_{1}^{2}-av_{1}^{3}+v_{2}-v_{3}+\beta \left(v_{1}-u_{1}\right),\\ {\dot{v}}_{2}=\phi_{3}v_{1}-dv_{1}^{2}-v_{2},\\ {\dot{v}}_{3}=r\left(s_{0}{\text{v}}_{1}-v_{3}\right), \end{array}$$

where *ϕ*_1_ = −3*ax*_01_^2^ + 2*bx*_01_, *ϕ*_2_ = −3*ax*_01_ + *b*, and *ϕ*_3_ = −2*dx*_01_. By letting

(5) $$\begin{array}{c} e_{1}=\frac{u_{1}-v_{1}}{2},\\ e_{2}=\frac{u_{2}-v_{2}}{2},\\ e_{3}=\frac{u_{3}-v_{3}}{2},\\ e_{4}=\frac{u_{1}+v_{1}}{2},\\ e_{5}=\frac{u_{2}+v_{2}}{2},\\ e_{6}=\frac{u_{3}+v_{3}}{2}, \end{array}$$

system (4) can be written as

(6) $$\begin{array}{c} {\dot{e}}_{1}=\left(2\beta +\phi_{1}\right)e_{1}+e_{2}-e_{3}+F_{1},\\ {\dot{e}}_{2}=\phi_{3}e_{1}-e_{2}+F_{2},\\ {\dot{e}}_{3}=r\left(s_{0}e_{1}-e_{3}\right),\\ {\dot{e}}_{4}=\phi_{1}e_{4}+e_{5}-e_{6}+F_{3},\\ {\dot{e}}_{5}=\phi_{3}e_{4}-e_{5}+F_{4},\\ {\dot{e}}_{6}=r\left(s_{0}e_{4}-e_{6}\right), \end{array}$$

where

(7) $$\begin{array}{c} F_{1}=-a\left(3e_{1}e_{4}^{2}+e_{1}^{3}\right)+2\phi_{2}e_{1}e_{4},\\ F_{2}=-2de_{1}e_{4},\\ F_{3}=-a\left(3e_{1}^{2}e_{4}+e_{4}^{3}\right)+\phi_{2}\left(e_{1}^{2}+e_{4}^{2}\right),\\ F_{4}=-d\left(e_{1}^{2}+e_{4}^{2}\right). \end{array}$$

If *e*_1,2,3_ → 0 for *t* → ∞, the two HR neurons will achieve synchronization. Consider the Laplace transform defined as follows:

(8) $$\begin{array}{c} {\hat{e}}_{i}=L\left[e_{i}\right]=\int_{0}^{+\infty }e_{i}e^{-st}dt,\\ e_{i}=L^{-1}\left[{\hat{e}}_{i}\right]=\frac{1}{2\pi i}\int_{\sigma -i\infty }^{\sigma +i\infty }{\hat{e}}_{i}\left(s\right)e^{st}ds,\;i=1,\cdots ,6. \end{array}$$

Taking the Laplace transforms of both sides of equation (6) and arranging them yield

(9) $$\begin{array}{c} \left(s-2\beta -\phi_{1}\right){\hat{e}}_{1}-{\hat{e}}_{2}+{\hat{e}}_{3}=e_{10}+{\hat{F}}_{1},\\ -\phi_{3}{\hat{e}}_{1}+\left(s+1\right){\hat{e}}_{2}=e_{20}+{\hat{F}}_{2},\\ -rs_{0}{\hat{e}}_{1}+\left(s+r\right){\hat{e}}_{3}=e_{30},\\ \left(s+\phi_{1}\right){\hat{e}}_{4}-{\hat{e}}_{5}+{\hat{e}}_{6}=e_{40}+{\hat{F}}_{3},\\ -\phi_{3}e_{4}+\left(s+1\right){\hat{e}}_{5}=e_{50}+{\hat{F}}_{4},\\ -rs_{0}{\hat{e}}_{4}+\left(s+r\right){\hat{e}}_{6}=e_{60}, \end{array}$$

where *e*_*i*0_, *i* = 1, ⋯, 6, are given initial values of system (6), and

(10) $$\begin{array}{c} {\hat{F}}_{j}=\int_{0}^{+\infty }\left[F_{j}\left(t\right)\right]e^{-st}dt,\;\;j=1,2,3,4. \end{array}$$

Since only *e*_1,2,3_ need to be considered, solving the first three equations in system (9) leads to

(11) $$\begin{array}{c} {\hat{e}}_{1}=\frac{g_{2}}{g_{1}}+\frac{\left[s^{2}+\left(r+1\right)s+r\right]{\hat{F}}_{1}}{g_{1}}+\frac{\left(s+r\right){\hat{F}}_{2}}{g_{1}},\\ {\hat{e}}_{2}=\frac{g_{3}}{g_{1}}+\frac{\left(r+s\right)\phi_{3}{\hat{F}}_{1}}{g_{1}}+\frac{\left[s^{2}+\left(r-2\beta -\phi_{1}\right)s+r\left(s_{0}-2\beta -\phi_{1}\right)\right]{\hat{F}}_{2}}{g_{1}},\\ {\hat{e}}_{3}=\frac{g_{4}}{g_{1}}+\frac{rs_{0}\left(s+1\right){\hat{F}}_{1}}{g_{1}}+\frac{rs_{0}{\hat{F}}_{2}}{g_{1}}, \end{array}$$

where

(12) $$\begin{array}{c} g_{1}=s^{3}-\left(2\beta -r+\phi_{1}-1\right)s^{2}-\left[2\left(r+1\right)\beta +r\left(\phi_{1}-1-s_{0}\right)+\phi_{1}+\phi_{3}\right]s-r\left(2\beta -s_{0}+\phi_{1}+\phi_{3}\right),\\ g_{2}=e_{10}s^{2}+\left[e_{10}\left(r+1\right)+e_{20}-e_{30}\right]s+r\left(e_{10}+e_{20}\right)-e_{30},\\ g_{3}=e_{20}s^{2}+\left[\phi_{3}e_{10}+\left(r-2\beta -\phi_{1}\right)e_{20}\right]s+e_{10}r\phi_{3}+r\left(s_{0}-2\beta -\phi_{1}\right)e_{20}-\phi_{3}e_{30},\\ g_{4}=e_{30}s^{2}+\left[rs_{0}e_{10}+\left(1-2\beta -\phi_{1}\right)e_{30}\right]s+rs_{0}\left(e_{10}+e_{20}\right)-\left(2\beta +\phi_{1}+\phi_{3}\right)e_{30}. \end{array}$$

From the convolution theorem, taking the inverse Laplace transform on both hands of equations in equation (11), one has

(13) $$\begin{array}{c} e_{1}=\Phi_{1}+\int_{0}^{t}\left[\Phi_{2}\left(t-\tau \right)F_{1}\left(\tau \right)+\Phi_{3}\left(t-\tau \right)F_{2}\left(\tau \right)\right]d\tau ,\\ e_{2}=\Phi_{4}+\int_{0}^{t}\left[\Phi_{5}\left(t-\tau \right)F_{1}\left(\tau \right)+\Phi_{6}\left(t-\tau \right)F_{2}\left(\tau \right)\right]d\tau ,\\ e_{3}=\Phi_{7}+\int_{0}^{t}\left[\Phi_{8}\left(t-\tau \right)F_{1}\left(\tau \right)+\Phi_{9}\left(t-\tau \right)F_{2}\left(\tau \right)\right]d\tau , \end{array}$$

where *Φ*_*i*_(*t*), *i* = 1, 2, ⋯, 9, denote the following inverse Laplace transforms, respectively,

(14) $$\begin{array}{c} \Phi_{1}\left(t\right)=L^{-1}\left[\frac{g_{2}}{g_{1}}\right],\\ \Phi_{2}\left(t\right)=L^{-1}\left[\frac{s^{2}+\left(r+1\right)s+r}{g_{1}}\right],\\ \;\Phi_{3}\left(t\right)=L^{-1}\left[\frac{s+r}{g_{1}}\right],\\ \Phi_{4}\left(t\right)=L^{-1}\left[\frac{g_{3}}{g_{1}}\right],\\ \;\Phi_{5}\left(t\right)=L^{-1}\left[\frac{\left(r+s\right)\phi_{3}}{g_{1}}\right],\\ \Phi_{6}\left(t\right)=L^{-1}\left[\frac{\left[s^{2}+\left(r-2\beta -\phi_{1}\right)s+r\left(s_{0}-2\beta -\phi_{1}\right)\right]}{g_{1}}\right],\\ \Phi_{7}\left(t\right)=L^{-1}\left[\frac{g_{4}}{g_{1}}\right],\\ \Phi_{8}\left(t\right)=L^{-1}\left[\frac{rs_{0}\left(s+1\right)}{g_{1}}\right],\\ \Phi_{9}\left(t\right)=L^{-1}\left[\frac{rs_{0}}{g_{1}}\right]. \end{array}$$

The kernels in *Φ*_*i*_(*t*), *i* = 1, 2, ⋯, 9, are true fractions which can be partitioned into the partial fraction expansions; therefore,

(15) $$\begin{array}{c} \Phi_{1,\cdots ,9}=\left\{\begin{array}{cc} L^{-1}\left[\frac{\xi_{1}}{s-s_{1}}+\frac{\xi_{2}}{s-s_{2}}+\frac{\xi_{3}}{s-s_{3}}\right]=\overset{3}{\underset{i=1}{\sum }}\xi_{i}e^{s_{i}t}, & s_{1}\neq s_{2}\neq s_{3},\\ L^{-1}\left[\frac{\xi_{1}}{{\left(s-s_{1}\right)}^{2}}+\frac{\xi_{2}}{s-s_{1}}+\frac{\xi_{3}}{s-s_{3}}\right]=\left(\xi_{1}t+\xi_{2}\right)e^{s_{1}t}+\xi_{3}e^{s_{3}t}, & s_{1}=s_{2}\neq s_{3},\\ L^{-1}\left[\frac{\xi_{1}}{{\left(s-s_{1}\right)}^{3}}+\frac{\xi_{2}}{{\left(s-s_{1}\right)}^{2}}+\frac{\xi_{3}}{s-s_{1}}\right]=\left(\frac{\xi_{1}}{2}t^{2}+\xi_{2}t+\xi_{3}\right)e^{s_{1}t}, & s_{1}=s_{2}=s_{3}, \end{array}\right. \end{array}$$

where *ξ*_*i*_ are undetermined constants and *s* = *s*_*i*_, *i* = 1, 2, 3, are the roots to the equation *g*_1_(*s*) = 0 (*g*_1_(*s*) is defined in equation (11)). If all the real parts of roots of *g*_1_(*s*) = 0 are negative, *Φ*_1,⋯,9_(*t*) → 0 when the time approaches to infinity. In this case, equation (13) becomes

(16) $$\begin{array}{c} e_{1}=\int_{0}^{t}\left[\Phi_{2}\left(t-\tau \right)F_{1}\left(\tau \right)+\Phi_{3}\left(t-\tau \right)F_{2}\left(\tau \right)\right]d\tau =\int_{0}^{t}\left[\Phi_{2}\left(t-\tau \right)\left(-a\left(3e_{4}{\left(\tau \right)}^{2}+e_{1}{\left(\tau \right)}^{2}\right)+2\phi_{2}e_{4}\left(\tau \right)\right)-2d\Phi_{3}\left(t-\tau \right)e_{4}\left(\tau \right)\right]e_{1}\left(\tau \right)d\tau ,\\ e_{2}=\int_{0}^{t}\left[\Phi_{5}\left(t-\tau \right)F_{1}\left(\tau \right)+\Phi_{6}\left(t-\tau \right)F_{2}\left(\tau \right)\right]d\tau =\int_{0}^{t}\left[\Phi_{5}\left(t-\tau \right)\left(-a\left(3e_{4}{\left(\tau \right)}^{2}+e_{1}{\left(\tau \right)}^{2}\right)+2\phi_{2}e_{4}\left(\tau \right)\right)-2d\Phi_{6}\left(t-\tau \right)e_{4}\left(\tau \right)\right]e_{1}\left(\tau \right)d\tau ,\\ e_{3}=\int_{0}^{t}\left[\Phi_{8}\left(t-\tau \right)F_{1}\left(\tau \right)+\Phi_{9}\left(t-\tau \right)F_{2}\left(\tau \right)\right]d\tau =\int_{0}^{t}\left[\Phi_{8}\left(t-\tau \right)\left(-a\left(3e_{4}{\left(\tau \right)}^{2}+e_{1}{\left(\tau \right)}^{2}\right)+2\phi_{2}e_{4}\left(\tau \right)\right)-2d\Phi_{9}\left(t-\tau \right)e_{4}\left(\tau \right)\right]e_{1}\left(\tau \right)d\tau . \end{array}$$

From the successive approximation method introduced in references [26, 27], *e*_1,2,3_ → 0 in equation (16) with time approaching to infinity if all the roots of *g*_1_(*s*) = 0 have negative real parts. *g*_1_(*s*) has the form of

(17) $$\begin{array}{c} g_{1}\left(s\right)=s^{3}+p_{1}s^{2}+p_{2}s+p_{3}. \end{array}$$

According to the Routh-Hurwitz criterion, 3 Hurwitz matrices using the coefficients *p*_*i*_, *i* = 1, 2, 3, of *g*_1_(*s*) are given by

(18) $$\begin{array}{c} H_{1}=\left[p_{1}\right],\\ H_{2}=\left[\begin{array}{cc} p_{1} & 1\\ 0 & p_{2} \end{array}\right],\\ \;H_{3}=\left[\begin{array}{ccc} p_{1} & 1 & 0\\ p_{3} & p_{2} & p_{1}\\ 0 & 0 & p_{3} \end{array}\right]. \end{array}$$

All of the roots of the polynomial *g*_1_(*s*) are negative or have negative real parts if and only if the determinants of all Hurwitz matrices are positive:

(19) $$\begin{array}{c} \det H_{j}>0,\;j=1,2,3. \end{array}$$

Since

(20) $$\begin{array}{c} \det H_{1}=p_{1}>0,\\ \det H_{2}=p_{1}p_{2}>0,\\ \;\det H_{3}=\left(p_{1}p_{2}-p_{3}\right)p_{3}>0, \end{array}$$

the necessary and sufficient condition that all the roots of *g*_1_(*s*) = 0 have negative real parts can be written as

(21) $$\begin{array}{c} 2\beta -r+\phi_{1}-1<0,\\ 2\left(r+1\right)\beta +r\left(\phi_{1}-1-s_{0}\right)+\phi_{1}+\phi_{3}<0,\\ r\left(2\beta -s_{0}+\phi_{1}+\phi_{3}\right)<0,\\ \left(2\beta -r+\phi_{1}-1\right)\left[2\left(r+1\right)\beta +r\left(\phi_{1}-1-s_{0}\right)+\phi_{1}+\phi_{3}\right]+r\left(2\beta -s_{0}+\phi_{1}+\phi_{3}\right)>0, \end{array}$$

where *ϕ*_1,3_ are defined in equation (4). Equation (21) just is the synchronization condition for the two coupled HR neurons in system (2).

## 3. Two HR Neurons with Nonlinear Coupling Functions

Consider two coupled HR neurons with a nonlinear coupling function

(22) $$\begin{array}{c} {\dot{x}}_{1}=y_{1}-ax_{1}^{3}+bx_{1}^{2}-z_{1}+I+\alpha \left(H\left(x_{1}\right)-H\left(x_{2}\right)\right),\\ {\dot{y}}_{1}=c-dx_{1}^{2}-y_{1}+\alpha \left(dx_{1}^{2}-dx_{2}^{2}\right),\\ {\dot{z}}_{1}=rs_{0}\left(x_{1}-x_{0}\right)-rz_{1},\\ {\dot{x}}_{2}=y_{2}-ax_{2}^{3}+bx_{2}^{2}-z_{2}+I+\alpha \left(H\left(x_{2}\right)-H\left(x_{1}\right)\right),\\ {\dot{y}}_{2}=c-dx_{2}^{2}-y_{2}+\alpha \left(dx_{2}^{2}-dx_{1}^{2}\right),\\ {\dot{z}}_{2}=rs_{0}\left(x_{2}-x_{0}\right)-rz_{2}, \end{array}$$

where *α* is the coupling strength and *H*(*x*) = *ax*^3^ − *bx*^2^ − *x*. Substituting equation (3) into equation (22) produces

(23) $$\begin{array}{c} {\dot{u}}_{1}=\phi_{1}u_{1}+\phi_{2}u_{1}^{2}-au_{1}^{3}+u_{2}-u_{3}+\alpha \left[a\left(u_{1}^{3}-v_{1}^{3}\right)-\phi_{2}\left(u_{1}^{2}-v_{1}^{2}\right)-\left(\phi_{1}+1\right)\left(u_{1}-v_{1}\right)\right],\\ {\dot{u}}_{2}=\phi_{3}u_{1}-du_{1}^{2}-u_{2}+\alpha \left[d\left(u_{1}^{2}-v_{1}^{2}\right)-\phi_{3}\left(u_{1}-v_{1}\right)\right],\\ {\dot{u}}_{3}=r\left(s_{0}u_{1}-u_{3}\right),\\ {\dot{v}}_{1}=\phi_{1}v_{1}+\phi_{2}v_{1}^{2}-av_{1}^{3}+v_{2}-v_{3}-\alpha \left[a\left(u_{1}^{3}-v_{1}^{3}\right)-\phi_{2}\left(u_{1}^{2}-v_{1}^{2}\right)-\left(\phi_{1}+1\right)\left(u_{1}-v_{1}\right)\right],\\ {\dot{v}}_{2}=\phi_{3}v_{1}-dv_{1}^{2}-v_{2}-\alpha \left[d\left(u_{1}^{2}-v_{1}^{2}\right)-\phi_{3}\left(u_{1}-v_{1}\right)\right],\\ {\dot{v}}_{3}=r\left(s_{0}v_{1}-v_{3}\right), \end{array}$$

where *ϕ*_1,2,3_ are defined in equation (4). Substituting equation (5) into equation (23) yields

(24) $$\begin{array}{c} {\dot{e}}_{1}=\left[-2\alpha \left(\phi_{1}+1\right)+\phi_{1}\right]e_{1}+e_{2}-e_{3}+F_{5},\\ {\dot{e}}_{2}=\left(1-2\alpha \right)\phi_{3}e_{1}-e_{2}+F_{6},\\ {\dot{e}}_{3}=r\left(s_{0}e_{1}-e_{3}\right),\\ {\dot{e}}_{4}=\phi_{1}e_{4}+e_{5}-e_{6}+F_{3},\\ {\dot{e}}_{5}=\phi_{3}e_{4}-e_{5}+F_{4},\\ {\dot{e}}_{6}=r\left(s_{0}e_{4}-e_{6}\right), \end{array}$$

where

(25) $$\begin{array}{c} F_{3}=-a\left(3e_{1}^{2}e_{4}+e_{4}^{3}\right)+\phi_{2}\left(e_{1}^{2}+e_{4}^{2}\right),\\ \;F_{4}=-d\left(e_{1}^{2}+e_{4}^{2}\right),\\ F_{5}=\left(2\alpha -1\right)\left[ae_{1}^{3}+3ae_{1}e_{4}^{2}-2\phi_{2}e_{1}e_{4}\right],\\ \;F_{6}=2d\left(2\alpha -1\right)e_{1}e_{4}. \end{array}$$

If *e*_1,2,3_ → 0, synchronization appears in equation (23). Taking the Laplace transforms on both sides of the first three equations in equation (24), one has

(26) $$\begin{array}{c} \left[s+2\alpha \left(\phi_{1}+1\right)-\phi_{1}\right]{\hat{e}}_{1}-{\hat{e}}_{2}+{\hat{e}}_{3}=e_{10}+{\hat{F}}_{5},\\ \left(2\alpha -1\right)\phi_{3}e_{1}+\left(s+1\right){\hat{e}}_{2}=e_{20}+{\hat{F}}_{6},\\ -rs_{0}{\hat{e}}_{1}+\left(s+r\right){\hat{e}}_{3}=e_{30}, \end{array}$$

where *e*_*i*0_, *i* = 1, 2, 3, are given initial values of system (24), and

(27) $$\begin{array}{c} {\hat{F}}_{j}=\int_{0}^{+\infty }\left[F_{j}\left(t\right)\right]e^{-st}dt,\;\;j=5,6. \end{array}$$

Solving equation (26) leads to

(28) $$\begin{array}{c} {\hat{e}}_{1}=\frac{h_{2}}{h_{1}}+\frac{\left[s^{2}+\left(r+1\right)s+r\right]{\hat{F}}_{5}}{h_{1}}+\frac{\left(s+r\right){\hat{F}}_{6}}{h_{1}},\\ {\hat{e}}_{2}=\frac{h_{3}}{h_{1}}+\frac{\left(r+s\right)\left(1-2\alpha \right)\phi_{3}{\hat{F}}_{5}}{h_{1}}-\frac{h_{4}{\hat{F}}_{6}}{h_{1}},\\ {\hat{e}}_{3}=\frac{h_{5}}{h_{1}}+\frac{rs_{0}\left(s+1\right){\hat{F}}_{5}}{h_{1}}+\frac{rs_{0}{\hat{F}}_{6}}{h_{1}}. \end{array}$$

where

(29) $$\begin{array}{c} h_{1}=s^{3}+\left(2\alpha \left(\phi_{1}+1\right)-\phi_{1}+r+1\right)s^{2}+\left[\left(2\alpha -1\right)\phi_{1}\left(r+1\right)+2\alpha \left(r+1+\phi_{3}\right)+r\left(s_{0}+1\right)-\phi_{3}\right]s+r\left[\left(2\alpha -1\right)\left(\phi_{1}+\phi_{3}\right)+s_{0}+2\alpha \right],\\ h_{2}=e_{10}s^{2}+\left[e_{10}\left(r+1\right)+e_{20}-e_{30}\right]s+r\left(e_{10}+e_{20}\right)-e_{30},\\ h_{3}=-e_{20}^{2}s^{2}+\left[e_{10}\left(2\alpha -1\right)\phi_{3}-\left(r+2\alpha \left(\phi_{1}+1\right)-\phi_{1}\right)e_{20}\right]s-e_{20}r\left[2\alpha \left(\phi_{1}+1\right)-\phi_{1}\right]+\left(e_{10}r-e_{30}\right)\left(2\alpha -1\right)\phi_{3}-rs_{0}e_{20},\\ h_{4}=s^{2}+\left(2\alpha \left(\phi_{1}+1\right)-\phi_{1}+r\right)s+r\left(2\alpha \left(\phi_{1}+1\right)-\phi_{1}+s_{0}\right),\\ h_{5}=e_{30}s^{2}+\left[rs_{0}e_{10}+\left(1+2\alpha \left(\phi_{1}+1\right)-\phi_{1}\right)e_{30}\right]s+rs_{0}\left(e_{10}+e_{20}\right)+\left[\left(2\alpha -1\right)\left(\phi_{1}+\phi_{3}\right)+2\alpha \right]e_{30}. \end{array}$$

Taking the inverse Laplace transform on both sides of the three equations in equation (28), one yields

(30) $$\begin{array}{c} e_{1}=\Psi_{1}\left(t\right)+\int_{0}^{t}\left[\Psi_{2}\left(t-\tau \right)F_{5}\left(\tau \right)+\Psi_{3}\left(t-\tau \right)F_{6}\left(\tau \right)\right]d\tau ,\\ e_{2}=\Psi_{4}\left(t\right)+\int_{0}^{t}\left[\Psi_{5}\left(t-\tau \right)F_{5}\left(\tau \right)-\Psi_{6}\left(t-\tau \right)F_{6}\left(\tau \right)\right]d\tau ,\\ e_{3}=\Psi_{7}\left(t\right)+\int_{0}^{t}\left[\Psi_{8}\left(t-\tau \right)F_{5}\left(\tau \right)+\Psi_{9}\left(t-\tau \right)F_{6}\left(\tau \right)\right]d\tau , \end{array}$$

where Ψ_1,⋯,9_(*t*) represent the following inverse Laplace transforms:

(31) $$\begin{array}{c} \Psi_{1}\left(t\right)=L^{-1}\left[\frac{h_{2}}{h_{1}}\right],\\ \;\Psi_{2}\left(t\right)=L^{-1}\left[\frac{s^{2}+\left(r+1\right)s+r}{h_{1}}\right],\\ \Psi_{3}\left(t\right)=L^{-1}\left[\frac{s+r}{h_{1}}\right],\\ \Psi_{4}\left(t\right)=L^{-1}\left[\frac{h_{3}}{h_{1}}\right],\\ \Psi_{5}\left(t\right)=L^{-1}\left[\frac{\left(r+s\right)\left(1-2\alpha \right)\phi_{3}}{h_{1}}\right],\\ \Psi_{6}\left(t\right)=L^{-1}\left[\frac{h_{4}}{h_{1}}\right],\\ \Psi_{7}\left(t\right)=L^{-1}\left[\frac{h_{5}}{h_{1}}\right],\\ \;\Psi_{8}\left(t\right)=L^{-1}\left[\frac{rs_{0}\left(s+1\right)}{h_{1}}\right],\\ \Psi_{9}\left(t\right)=L^{-1}\left[\frac{rs_{0}}{h_{1}}\right]. \end{array}$$

Furthermore, Ψ_1,⋯,9_ can be expressed as the following forms:

(32) $$\begin{array}{c} \Psi_{1,\cdots ,9}=\left\{\begin{array}{cc} L^{-1}\left[\frac{\eta_{1}}{s-s_{1}}+\frac{\eta_{2}}{s-s_{2}}+\frac{\eta_{3}}{s-s_{3}}\right]=\overset{3}{\underset{i=1}{\sum }}\eta_{i}e^{s_{i}t}, & s_{1}\neq s_{2}\neq s_{3},\\ L^{-1}\left[\frac{\eta_{1}}{{\left(s-s_{1}\right)}^{2}}+\frac{\eta_{2}}{s-s_{1}}+\frac{\eta_{3}}{s-s_{3}}\right]=\left(\eta_{1}t+\eta_{2}\right)e^{s_{1}t}+\eta_{3}e^{s_{3}t}, & s_{1}=s_{2}\neq s_{3},\\ L^{-1}\left[\frac{\eta_{1}}{{\left(s-s_{1}\right)}^{3}}+\frac{\eta_{2}}{{\left(s-s_{1}\right)}^{2}}+\frac{\eta_{3}}{s-s_{1}}\right]=\left(\frac{\eta_{1}}{2}t^{2}+\eta_{2}t+\eta_{3}\right)e^{s_{1}t}, & s_{1}=s_{2}=s_{3}, \end{array}\right. \end{array}$$

where *η*_*i*_ are undetermined constants and *s* = *s*_*i*_, *i* = 1, 2, 3, are the roots to the equation *h*_1_(*s*) = 0 (*h*_1_(*s*) is defined in equation (28)). From the analysis for the case of a linear coupling function in the previous section, if Ψ_1,⋯,9_ → 0 when the time approaches to infinity, *e*_1,2,3_ → 0 in equation (24) will be achieved, which means that all roots of *h*_1_(*s*) = 0 should have negative real parts. Based on the Routh-Hurwitz criterion, the synchronization condition in system (23) can be written as

(33) $$\begin{array}{c} 2\alpha \left(\phi_{1}+1\right)-\phi_{1}+r+1>0,\\ \left(2\alpha -1\right)\phi_{1}\left(r+1\right)+2\alpha \left(r+1+\phi_{3}\right)+r\left(s_{0}+1\right)-\phi_{3}>0,\\ r\left[\left(2\alpha -1\right)\left(\phi_{1}+\phi_{3}\right)+s_{0}+2\alpha \right]>0,\\ \left[2\alpha \left(\phi_{1}+1\right)-\phi_{1}+r+1\right]\left[\left(2\alpha -1\right)\phi_{1}\left(r+1\right)+2\alpha \left(r+1+\phi_{3}\right)+r\left(s_{0}+1\right)-\phi_{3}\right]-r\left[\left(2\alpha -1\right)\left(\phi_{1}+\phi_{3}\right)+s_{0}+2\alpha \right]>0. \end{array}$$

## 4. Numerical Simulations

**Figure 1:**
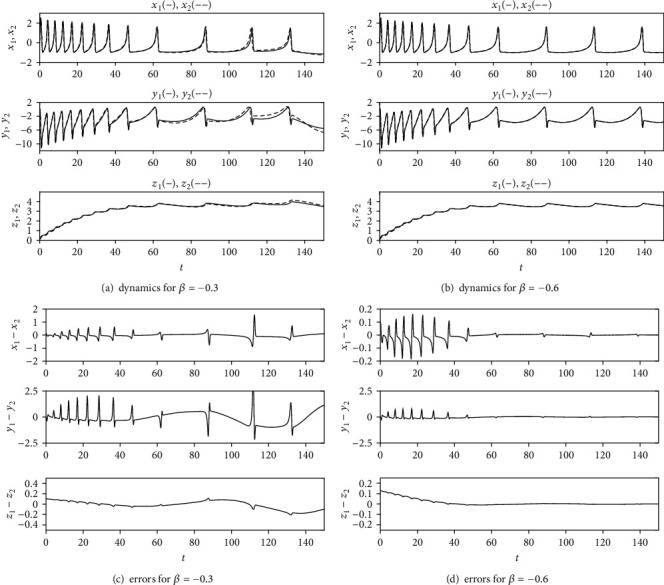
The dynamics of system (2) with *r* = 0.02 and *I* = 3.6: (a) *β* = −0.3 and (b) *β* = −0.6. Synchronization errors: (c) *β* = −0.3 and (d) *β* = −0.6. The initial conditions are *x*_1_(0) = 0.2, *y*_1_(0) = 0.1, *z*_1_(0) = 0.2, *x*_2_(0) = 0.1, *y*_2_(0) = 0.1, and *z*_2_(0) = 0.1.

In this section, some numerical simulations are performed to illustrate the correctness of the synchronization criteria derived in previous sections. Here, *a* = 1.0, *b* = 3.0, *c* = 1.0, *d* = 5.0, *s*_0_ = 4, and *x*_0_ = −1.60. First, the case of the linear function, which is system (2), is considered. If *r* = 0.02 and *I* = 3.6, the two HR neurons in system (2) with *β* = 0 exhibit periodic spikes. From equation (21), the synchronization condition for system (2) is *β* < −0.55. To demonstrate the validity of the coupling strength, *β* = −0.6 and *β* = −0.3 are chosen to carry out the numerical simulations of system (2), respectively. The initial conditions are taken as *x*_1_(0) = 0.2, *y*_1_(0) = 0.1, *z*_1_(0) = 0.2, *x*_2_(0) = 0.1, *y*_2_(0) = 0.1, and *z*_2_(0) = 0.1. The numerical results are presented in Figure 1, which demonstrates the effectiveness of the synchronization condition (21).

**Figure 2:**
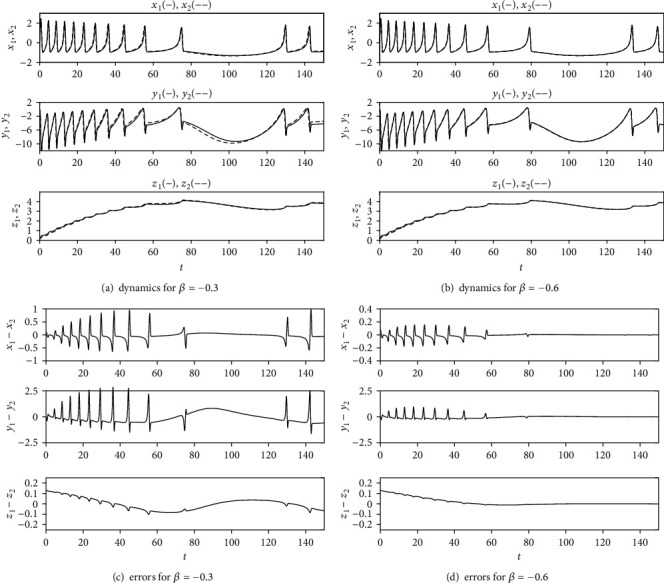
The dynamics of system (2) with *r* = 0.013 and *I* = 3.0: (a) *β* = −0.3 and (b) *β* = −0.6. Synchronization errors: (c) *β* = −0.3 and (d) *β* = −0.6. The initial conditions are *x*_1_(0) = 0.2, *y*_1_(0) = 0.1, *z*_1_(0) = 0.2, *x*_2_(0) = 0.1, *y*_2_(0) = 0.1, and *z*_2_(0) = 0.1.

When *r* = 0.013 and *I* = 3, the two HR neurons in system (2) with *β* = 0 exhibit burst-spike chaotic behavior. According to equation (21), the synchronization condition for system (2) is *β* < −0.56. To demonstrate the validity of the coupling strength, *β* = −0.6 and *β* = −0.3 are taken to perform the numerical simulations of system (2), respectively. The initial conditions were kept the same as those in Figure 1. The results are presented in Figure 2, which illustrates the correctness of the synchronization condition (21).

For the case of the nonlinear coupling function, let *x*_0_ = −1.56, *r* = 0.006, *I* = 3.0, and other parameters keep unchanged. According to [22], based on the conditional Lyapunov exponent method, the synchronization condition is approximately given as *α* ∈ [0.265,0.61]. From criterion (33), the synchronization condition is [0.275,0.7]. Therefore, the synchronization criterion proposed in this paper is valid.

## 5. Conclusion

In this paper, the synchronization between two HR neurons with linear and nonlinear coupling functions is investigated by using the Laplace transform and the convolution theorem. Different from other researchers, the synchronization error system can be expressed in its integral form, which is more convenient to analyze. The synchronization problem is ultimately converted into the stability problem of roots to a nonlinear algebraic equation. Then, an analytical criterion for synchronization in the two HR neurons can be given by using the Routh-Hurwitz criterion. Numerical simulations show that the synchronization criterion derived in this paper is effective, regardless of the periodic spikes or burst-spike chaotic behavior of the two HR neurons. Furthermore, our analytical results have almost the same accuracy as the conditional Lyapunov method. Since the calculation quantities are very small, the approach presented in this paper is easy to be developed to study synchronization between a large number of HR neurons.

## Data Availability

No data were used to support this study.
